# Recurrent anterior uveitis and subsequent incidence of ankylosing spondylitis: a nationwide cohort study from 2002 to 2013

**DOI:** 10.1186/s13075-018-1522-2

**Published:** 2018-02-07

**Authors:** Baek-Lok Oh, Jeong Seok Lee, Eun Young Lee, Hee Young Lee, Hyeong Gon Yu

**Affiliations:** 1 Department of Ophthalmology, Armed Forces Capital Hospital, Seongnam, Gyeonggi-do South Korea; 2 Division of Rheumatology, Department of Internal Medicine, Seoul National University College of Medicine, Seoul National University Hospital, Seoul, South Korea; 3 Center for Preventive Medicine and Public Health, Seoul National University Bundang Hospital, Seongnam, Gyeonggi-do South Korea; 4 Department of Ophthalmology, Seoul National University College of Medicine, Seoul National University Hospital, Seoul, South Korea

**Keywords:** Ankylosing spondylitis, Uveitis, Recurrence, Incidence rate

## Abstract

**Background:**

Anterior uveitis is the most common extra-articular manifestation of ankylosing spondylitis (AS). AS-related anterior uveitis frequently presents as acute, recurrent iridocyclitis; therefore, AS is often initially suspected by an ophthalmologist, not by a rheumatologist. In this study, we aimed to investigate the relationship between the recurrence of anterior uveitis and the subsequent incidence of AS.

**Methods:**

From a national sample cohort, 10,483 patients with new-onset uveitis between 2004 and 2013 and 52415 matched control subjects who had never experienced uveitis were selected. Among the patients with new-onset uveitis, a subpopulation of patients with recurrent uveitis, defined as a minimum 120-day reported interval between consecutive claims of uveitis (based on diagnostic codes) treated with local or systemic steroids or immune-modulating drugs, was identified. The incidence rates of AS were calculated according to the number of episodes of uveitis, and the incidence rate ratios (IRRs) were derived on the basis of the incidence rate in the control group.

**Results:**

The incidence rate per 100,000 person-years of AS after the first uveitis episode was 121.5, whereas the incidence in the control group was 16.9 (IRR, 7.40; 95% CI, 4.99–10.98); after the second uveitis episode, the IRR increased to 17.71 (95% CI, 10.44–30.06). In male and female patients with recurrent uveitis, the incidence rates of AS were 284.1 and 268.7 per 100,000 person-years, respectively. In patients aged under 40 years, the IRR of the recurrent uveitis group was 46.78 (95% CI, 19.61–111.61). In patients aged over 59 years, AS incidence in the recurrent uveitis group did not differ from that in the control group.

**Conclusions:**

The risk of subsequent AS increased with the number of episodes of anterior uveitis. This quantitative evidence could contribute to the establishment of a rationale for ancillary workup for possible systemic associations in patients with recurrent uveitis.

**Electronic supplementary material:**

The online version of this article (10.1186/s13075-018-1522-2) contains supplementary material, which is available to authorized users.

## Background

Uveitis is one of the important clinical signs of systemic inflammatory diseases. Identification of systemic associations in patients with uveitis is important not only to determine the etiology of ocular inflammation and improve visual prognosis but also to improve the outcome of the systemic conditions themselves through a targeted therapeutic approach. Bilateral occurrence, granulomatous appearance, or recurrence of anterior uveitis, or posterior extension/involvement of uveitis, might be a clue to a possible systemic association, and ancillary tests should be considered in such cases [1].

Ankylosing spondylitis (AS) is a chronic inflammatory disease with axial spondyloarthritis whose classification criteria include the presence of anterior uveitis [2]. A recent meta-analysis demonstrated that anterior uveitis was the most common extra-articular manifestation of AS, with a prevalence of 25.8% [3]. A rough quantitative estimate of the association between anterior uveitis and the subsequent risk of AS can be obtained from previous studies. For example, researchers in a previous study found that the prevalence of AS in the general population varied from < 0.01% to 1.8% globally [4], whereas other studies have shown that AS was prevalent in 4.7–9.3% of patients with anterior uveitis visiting uveitis referral centers [5, 6]. However, these results do not provide quantitative information regarding the risk of subsequent AS in patients with uveitis in the general population.

Therefore, in this study, we investigated the relationship between the recurrence of uveitis and the incidence of subsequent AS in the Korean population by using data from a 12-year national sample cohort. Furthermore, we provide partial quantitative evidence regarding additional workup, including laboratory tests and radiographic imaging, in patients with recurrent anterior uveitis.

## Methods

### Source population

The present study was exempt from ethical review by the institutional review board because the data originated from de-identified secondary data released by the National Health Insurance Service (NHIS) for research purposes. The NHIS is a compulsory national health insurance that provides universal coverage for almost all residents in Korea. For reimbursement, all healthcare providers submit insurance claims to the NHIS with data including information on demographics, diagnostic codes, prescription records, and healthcare facilities. Therefore, these data have a centralized database structure. The dataset used in this study is an NHIS national sample cohort comprising claims submitted by healthcare providers from 2002 through 2013. The total population of this cohort consisted of 1,025,340 nationally representative random subjects, which represents approximately 2.2% of the total number of patients enrolled in NHIS in 2002. Patients were selected using the stratified random sampling method with 1476 strata according to sex, age, and income level [7].

### Study variables

We identified cases of anterior uveitis by identifying specific International Classification of Diseases, Tenth Revision (ICD-10), diagnostic codes (ICD-10 codes H20, H20.0, H20.00, H20.01, H20.04, H20.09, H20.1, H20.8, and H20.9) between January 1, 2002, and December 31, 2013. To specifically include cases of anterior uveitis, we selected patients who were treated using steroids or immunosuppressive agents (Additional file 1: Table S1) on the same day they were diagnosed with a diagnostic code of anterior uveitis. We washed out the first 2 years (2002–2003) for newly detected cases. Among patients diagnosed with AS, those who were diagnosed with anterior uveitis after their AS diagnosis, based on the visitation date, were excluded. Identification of patients with AS using this procedure was based on any claim with a diagnostic code of AS (ICD-10 codes M45.0–45.9). Ultimately, 10,483 patients with incident anterior uveitis in the years 2004–2013 were selected as the study group. The day of the initial diagnosis of uveitis, with treatment, was designated as the index date of the first uveitis episode. We matched up to five control subjects without uveitis from the population cohort to every patient with uveitis according to age, sex, household income level, and the year of uveitis diagnosis. The first day of the year used to match patients with uveitis and control subjects was designated as the index date for the control patients.

**Fig. 1:**
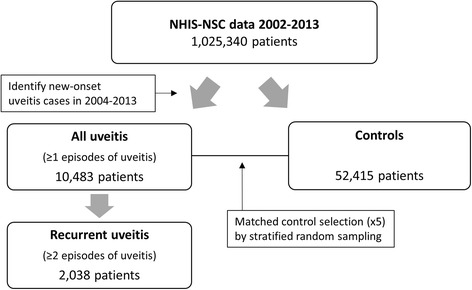
Flowchart depicting the classification of patients into the “all uveitis,” recurrent uveitis, and control groups. *NHIS-NSC* National Health Insurance Service - National Selection Cohort

According to the Standardization of Uveitis Nomenclature Working Group [8], the term *recurrent uveitis* describes episodes of uveitis separated by at least 3 months of inactivity without treatment. With this claim-based dataset, information about uveitis activity could not be retrieved, and we assumed that uveitis with a prescription of steroid or immunosuppressive agent was active. Details of the prescription provided the duration for which systemic medications, such as oral medication or injection, were supposed to be administered. However, we could not determine the duration for which topical medications, such as steroid ophthalmic solutions, were applied by the patients based on the prescription data. Considering that a bottle of topical steroid is usually used within 1 month, we defined uveitis recurrence as claims of anterior uveitis with treatment separated by at least 120 days. In the uveitis group, patients who experienced recurrence at least once were designated as the recurrent uveitis group and the day of first recurrence was designated as the index date for the second uveitis episode (Fig. 1).

Subsequent AS cases were operationally defined as those involving patients with diagnostic codes of AS (ICD-10 codes M45.0–45.9) claimed at a general hospital or referral center. Any patients who had also been diagnosed with systemic lupus erythematosus (ICD-10 code M32.0) or rheumatoid arthritis (RA; ICD-10 code M05.0 or M06.0) were excluded. Patients diagnosed with AS before 2004 were also excluded to ensure that the AS group included only patients with new episodes. The day of the first visit for AS was designated as the index date. A brief data exploration was conducted to obtain the overall prevalence and sex-specific distribution of AS to confirm the validity of the operational definition. Detailed information of the patients is available in Additional file 2: Table S2.

To aid in the interpretation of the results of this study, we performed an additional analysis with disease controls; patients with RA (ICD-10 codes M05.0–05.9, M06.0–06.9) and herniated intervertebral disc (HIVD; ICD-10 code M51.2) were included as the disease control subjects. The above-mentioned analysis regarding AS was repeated for RA and HIVD.

### Statistical analysis

The primary outcome of this study was the incidence rate ratio (IRR) of AS according to the number of episodes of uveitis: control group (no uveitis), “all uveitis” group (at least one uveitis episode), and recurrent uveitis group (at least two uveitis episodes). The time period was tracked from the index date of each group to (1) the day of AS diagnosis, if any; (2) the day of death, if any; or (3) the last day of the cohort analysis. Incidence rates were calculated as the number of people in whom AS developed after each uveitis episode divided by the total person-time at risk during the study period. The IRR and its 95% CI for AS were calculated using Poisson regression models (i.e., generalized linear models with a Poisson log-linear link function and person-years as the offset variable) based on the incidence rate of AS in the control group. Potential confounding variables such as age, sex, level of income, and year at diagnosis of uveitis episode were included in this model. Separate subgroup analyses with sex and age were performed. Age was stratified into three subgroups: < 40, 40–59, and > 59 years. Statistical analyses were performed using SAS software version 9.3 (SAS Inc., Cary, NC, USA) and R version 3.3.2 (R Foundation for Statistical Computing, Vienna, Austria; http://www.r-project.org). The cutoff for statistical significance was set at *P* < 0.05.

## Results

In the present study, 10,483 patients with newly developed anterior uveitis were identified during the period 2004–2013; the average incidence of anterior uveitis was 102.2 per 100,000 person-years. The male/female ratio was 1.14. Table 1 shows the baseline sociodemographic composition of the study population for the three groups: the control group, the “all uveitis” group, and the recurrent uveitis group. We examined 10,483 patients with uveitis and 52,415 sociodemographically matched control subjects. Among the 10,483 patients with uveitis, 2050 (19.6%) experienced at least one or more episodes of uveitis. Excluding the 12 patients who were diagnosed with AS before the second uveitis episode, the recurrent uveitis group comprised 2038 patients. AS developed in 0.08% of the control group subjects, in 0.54% of the patients in the “all uveitis” group, and in 1.03% of those in the recurrent uveitis group (*P* < 0.001). Age, sex, household income, and year of the index date were similar between the control group and uveitis group because these variables were used for matched sampling. The year of the index date in the recurrent uveitis group was later than that in the “all uveitis” group, because the first attacks preceded the second. The median age of the recurrent uveitis group was less than that of the “all uveitis” group (47.5 years vs 52.5 years). For patients with at least one episode of uveitis, the median number of uveitis episodes before AS development was 2 (IQR, 1–3), and the median interval between the first episode of uveitis and AS development was 103.3 weeks (interquartile range, 22.6–214.7 weeks).

**Table 1:** Baseline demographics of patients with uveitis and matched control subjects

	Control subjects (*n* = 52,415)	All uveitis (*n* = 10,483)	Recurrent uveitis (*n* = 2038)	*P* value
Ankylosing spondylitis				< 0.001
No	52,371 (99.9%)	10,426 (99.5%)	2017 (99.0%)	
Yes	44 (0.08%)	57 (0.54%)	21 (1.03%)	
Sex				0.983
Male	28,015 (53.4%)	5603 (53.4%)	1085 (53.2%)	
Female	24,400 (46.6%)	4880 (46.6%)	953 (46.8%)	
Age at diagnosis (years)				< 0.001
< 30	9030 (17.2%)	1806 (17.2%)	267 (13.1%)	
30–39	7535 (14.4%)	1507 (14.4%)	360 (17.7%)	
40–49	8980 (17.1%)	1796 (17.1%)	406 (19.9%)	
50–59	8030 (15.3%)	1606 (15.3%)	350 (17.2%)	
60–69	8380 (16.0%)	1676 (16.0%)	322 (15.8%)	
≥ 70	10,460 (20.0%)	2092 (20.0%)	333 (16.3%)	
Household income				> 0.999
First quintile	8760 (16.7%)	1752 (16.7%)	350 (17.2%)	
Second quintile	7510 (14.3%)	1502 (14.3%)	295 (14.5%)	
Third quintile	9650 (18.4%)	1930 (18.4%)	369 (18.1%)	
Fourth quintile	11,200 (21.4%)	2240 (21.4%)	437 (21.4%)	
Fifth quintile (high)	15,295 (29.2%)	3059 (29.2%)	587 (28.8%)	
Year of diagnosis				< 0.001
2004–2005	9020 (17.2%)	1804 (17.2%)	156 (7.67%)	
2006–2007	8980 (17.1%)	1796 (17.1%)	313 (15.4%)	
2008–2009	9920 (18.9%)	1984 (18.9%)	390 (19.2%)	
2010–2011	12,440 (23.7%)	2488 (23.7%)	559 (27.5%)	
2012–2013	12,055 (23.0%)	2411 (23.0%)	617 (30.3%)	

**Fig. 2:**
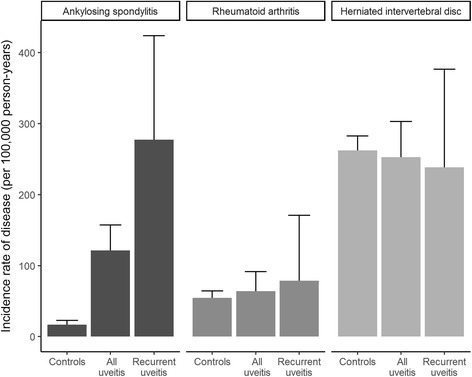
Incidence of ankylosing spondylitis, rheumatoid arthritis, and herniated intervertebral disc according to uveitis episode numbers

Incidence rates per 100,000 person-years of AS in patients in the “all uveitis” group, recurrent uveitis group, and control group are shown in Fig. 2. The IRRs corresponding to each condition are summarized in Table 2. The incidence rate per 100,000 person-years of AS was 16.9 (95% CI, 12.3–22.7) in the control group. Among patients who experienced a uveitis episode at least once, the AS incidence rate increased to 121.5/100,000 person-years (95% CI, 92.0–157.4). The incidence rates rose to 277.3 (95% CI, 171.6–423.8) in patients who experienced more than two episodes of uveitis. As disease controls, the RA and HIVD incidences did not show a correlation with the occurrence or recurrence of uveitis.

**Table 2:** Incidence rate ratio of diseases, along with number of episodes of uveitis

Disease	Group	No. of diseases	Person-years	IR	IRR (95% CI)	*P* value
AS	Control subjects	44	52,415	16.9	1 (reference)	
	All uveitis	57	10,483	121.5	7.40 (4.99–10.98)	< 0.001
	Recurrent uveitis	21	2038	277.3	17.71 (10.44–30.06)	< 0.001
RA	Control subjects	141	52,241	54.5	1 (reference)	
	All uveitis	30	10,439	64.0	1.16 (0.78–1.71)	0.473
	Recurrent uveitis	6	2038	78.6	1.45 (0.64–3.28)	0.377
HIVD	Control subjects	671	51,833	262.2	1 (reference)	
	All uveitis	117	10,329	252.9	0.97 (0.80–1.18)	0.757
	Recurrent uveitis	18	2019	238.4	0.93 (0.58–1.48)	0.750

*Abbreviations: AS* Ankylosing spondylitis, *HIVD* Herniated intervertebral disc, *IR* Incidence rate per 100,000 person-years, *IRR* Incidence rate ratio, *RA* Rheumatoid arthritis

**Fig. 3:**
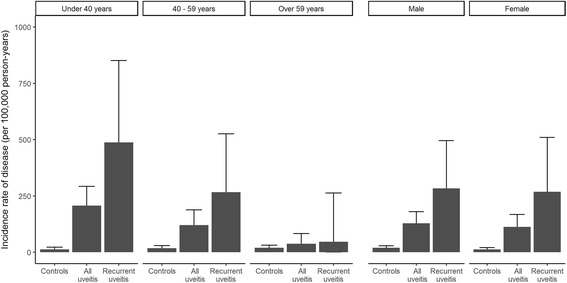
Incidence of ankylosing spondylitis according to age, sex, and uveitis episode numbers

Subgroup analysis for age and sex are provided in Table 3 and Fig. 3. Among the controls, AS occurred predominantly in males (20.4 vs 12.7 per 100,000 person-years). However, among patients with recurrent uveitis, the predilection for male sex was not prominent (284.1 vs 268.7 per 100,000 person-years). In patients aged under 40 years, the IRR for the recurrent uveitis group was 46.78 (95% CI, 19.61–111.61). In contrast, AS incidence in the recurrent uveitis groups aged over 59 years did not differ from that of the controls.

**Table 3:** Incidence rate ratio of diseases along with sex and number of episodes of uveitis

Subgroup	Group	No. of AS	Person-years	IR	IRR (95% CI)	*P* value
By age						
< 40 years	Control subjects	11	86,359	12.7	1 (reference)	
	All uveitis	32	15,430	207.4	17.64 (8.87–35.09)	< 0.001
	Recurrent uveitis	12	2461	487.5	46.78 (19.61–111.61)	< 0.001
40–59 years	Control subjects	16	87,723	18.2	1 (reference)	
	All uveitis	19	15,745	120.7	6.70 (3.44–13.06)	< 0.001
	Recurrent uveitis	8	2994	267.2	15.12 (6.41–35.65)	< 0.001
> 59 years	Control subjects	17	85,785	19.8	1 (reference)	
	All uveitis	6	15,739	38.1	1.90 (0.75–4.81)	0.178
	Recurrent uveitis	1	2119	47.2	2.46 (0.33–18.53)	0.382
By sex						
Male	Control subjects	29	142,124	20.4	1 (reference)	
	All uveitis	33	25,678	128.5	6.34 (3.84–10.44)	< 0.001
	Recurrent uveitis	12	4224	284.1	14.38 (7.28–28.41)	< 0.001
Female	Control subjects	15	117,744	12.7	1 (reference)	
	All uveitis	24	21,235	113.0	9.54 (4.99–18.24)	< 0.001
	Recurrent uveitis	9	3350	268.7	23.61 (10.05–55.47)	< 0.001

*Abbreviations: AS* Ankylosing spondylitis, *IR* Incidence rate per 100,000 person-years, *IRR* Incidence rate ratio

## Discussion

In this retrospective, community-based cohort study, we observed that the incidence rate of AS increased with recurrence of episodes of uveitis: 7.4-fold after one uveitis episode and 17.7-fold after two episodes. Furthermore, age and sex had an impact on the association between the number of uveitis episodes and subsequent AS incidence among the groups.

The methodology used for detecting the recurrence of episodes of uveitis is the main highlight of the present study. We had to identify whether a patient with uveitis had a 90-day inactive phase without uveitis treatment during sequential visits with a diagnostic code of uveitis. Considering that claims with a uveitis diagnostic code could not guarantee an active episode, claims with corresponding diagnostic codes and a prescription code for either steroid or immunosuppressive agents on the same day as the diagnosis were designated active uveitis cases. Thereafter, we defined recurrence of uveitis as cases where the time interval between two episodes of active anterior uveitis was more than 120 days, assuming that a bottle of topical steroid is usually used for 1 month. However, it was possible that the actual duration of application of ophthalmic solutions was less than 1 month, and our criterion of 120 days for defining uveitis recurrence was too long. To confirm whether our study results based on this criterion were stable and robust, we recalculated the primary outcome of the study with different values for time of recurrence interval and found that the results did not vary substantially (Additional file 3: Table S3). Thus, although the criterion for the time interval between claims for identifying recurrence was not indisputably definite, this was estimated to have no significant impact on the results of this study.

We performed additional analyses with disease controls to support the methodology used in this study because the results of a claim-based cohort study could potentially be biased by the increasing annual use of healthcare, especially in chronic diseases, or by the availability of healthcare for only certain diseases. RA is known to be a prevalent inflammatory arthritis that is not associated with uveitis [9]. In our study, RA did not show a correlation with uveitis, either as a single episode or recurrent. HIVD is a representative spondylopathy that is presumably not related to uveitis. If a patient with uveitis had a symptom of spondylopathy and thus the possibility of the patient visiting a spine clinic, the HIVD detection rate hence would have been higher. However, the detected occurrence of HIVD among patients with uveitis was not different from that in the control subjects. On the basis of these results, we assumed that our method of assessing the risk of systemic association in patients with uveitis was not significantly biased.

Male predominance is a well-known characteristic of AS. However, it was not prominent in patients with recurrent uveitis. Therefore, in managing female patients with recurrent uveitis, the possibility of the AS association should not be underestimated. The age-specific subgroup analysis showed that recurrent uveitis was associated with a substantially elevated risk of AS development in younger patients, and additional workup for uveitis should be considered to investigate any systemic association, including AS.

Although a claim data-based epidemiological analysis is advantageous for studying a rare disease and its associations with other diseases, there are certain limitations. First, the diagnoses of uveitis and AS were not clinically confirmed individually according to strict classification criteria, and overestimation or underestimation of the presence of diseases could have occurred. To restrict the overestimation of incident cases, we added the precondition that the identification of a uveitis episode required the simultaneous prescription of a local or systemic steroid or immunosuppressive agent and excluded patients with AS who had diagnostic codes of systemic lupus erythematosus or RA. Underestimation was also possible if a patient with a certain disease did not use the NHIS. In addition, systemic treatment for anterior uveitis might suppress or mask the incidence of subsequent AS. However, because anterior uveitis can usually be managed with topical and oral steroid administration for a limited period, it is unlikely that this treatment can suppress the onset of subsequent AS itself. Second, some potentially useful information was unavailable because medical records and laboratory test results of each patient were not included in the claim data; detailed characteristics of anterior uveitis (e.g., grade of inflammation, granulomatous or nongranulomatous), details of AS manifestation at onset, human leukocyte antigen B27 positivity, and levels of C-reactive protein and acute-phase reactant at the onset of uveitis or AS were not considered. This study would have been more informative if these variables could have been included in the regression model and if the analysis had indicated which variables were valuable in predicting subsequent AS occurrence in patients with uveitis, if any. Inactivity and recurrence of uveitis were also estimated indirectly by the type and schedule of prescribed medication. Although there are some intrinsic limitations of the insurance claim data, the general trend of the association between AS and recurrent uveitis was still obvious.

## Conclusions

We evaluated the risk of subsequent AS development after recurrent uveitis among Korean patients during a 10-year follow-up period. The incidence of AS increased proportionally with the number of episodes of uveitis. The estimated incidence of AS increased 7.4- and 17.7-fold after the first and second episodes of uveitis, respectively. This study provides a rationale for ancillary workup for possible systemic associations in patients with recurrent uveitis and emphasizes the importance of multidisciplinary collaboration in the diagnosis and management of AS.

## Additional files

Additional file 1: Table S1. List of steroids and immunosuppressive agents used for defining anterior uveitis. (DOCX 13 kb)

Additional file 2: Table S2. Detailed information on systemic diseases in the study cohort. (DOCX 14 kb)

Additional file 3: Table S3. Estimation of incidence rate ratio of ankylosing spondylitis in recurrent uveitis along with the time interval used for defining uveitis recurrence. (DOCX 13 kb)

## Abbreviations

AS: Ankylosing spondylitis
HIVD: Herniated intervertebral disc
ICD-10: International Classification of Diseases, Tenth Revision
IR: Incidence rate
IRR: Incidence rate ratio
NSC: National Sample Cohort
NHIS: National Health Insurance Service
RA: Rheumatoid arthritis
