# Supplementary material for: Recurrent anterior uveitis and subsequent incidence of ankylosing spondylitis: a nationwide cohort study from 2002 to 2013
Source: Arthritis Res Ther. 2018 Feb 7;20:22. doi: 10.1186/s13075-018-1522-2 (PMC5804077; doi:10.1186/s13075-018-1522-2)
Supplement: Supplementary file 1 — List of steroids and immunosuppressive agents used for defining anterior uveitis. (DOCX 13 kb) [file 13075_2018_1522_MOESM1_ESM.docx]

**Additional file 1: Table S1. List of steroid and immunosuppressive agents used for defining anterior uveitis.**

| Active ingredient of drug | Route |
| --- | --- |
| Adalimumab | Injection |
| Azathioprine | Oral medication |
| Betamethasone | Oral medication |
| Betamethasone | Injection |
| Cyclophosphamide | Injection |
| Cyclosporine | Oral medication |
| Cyclosporine | Injection |
| Deflazacort | Oral medication |
| Dexamethasone | Oral medication |
| Dexamethasone | Injection |
| Etanercept | Injection |
| Fludrocortisone | Oral medication |
| Fluorometholone | Topical medication |
| Hydrocortisone | Oral medication |
| Hydrocortisone | Injection |
| Infliximab | Injection |
| Methotrexate | Injection |
| Methylprednisolone | Oral medication |
| Methylprednisolone | Injection |
| Mycophenolate | Oral medication |
| Prednisolone | Oral medication |
| Prednisolone | Topical medication |
| Prednisolone | Injection |
| Rimexolone | Topical medication |
| Rituximab | Injection |
| Triamcinolone | Oral medication |
| Triamcinolone | Injection |
