# Supplementary material for: Recurrent anterior uveitis and subsequent incidence of ankylosing spondylitis: a nationwide cohort study from 2002 to 2013
Source: Arthritis Res Ther. 2018 Feb 7;20:22. doi: 10.1186/s13075-018-1522-2 (PMC5804077; doi:10.1186/s13075-018-1522-2)
Supplement: Supplementary file 2 — Detailed information on systemic diseases in the study cohort. (DOCX 14 kb) [file 13075_2018_1522_MOESM2_ESM.docx]

**Additional file 2: Table S2.** **Detailed information on systemic diseases in the study cohort.**

|  | AS^*^ | RA^†^ | HIVD^‡^ |
| --- | --- | --- | --- |
| Prevalent cases (2002-2013) | 1737 | 4959 | 20040 |
| Prevalence | 0.17% | 0.48% | 1.95% |
| Prevalence in references | 0.007-1.7% | 0.1-5% | 1-3% |
| Incidence cases (2004-2013) | 1339 | 3748 | 17121 |
| Incidence rate^§^ | 13.4 | 36.55 | 166.98 |
| By sex |  |  |  |
| Male | 775 | 902 | 7368 |
| Female | 564 | 2846 | 9753 |
| By age (years) |  |  |  |
| Median | 42.5 | 57.5 | 52.5 |
| Peak | 37.5 | 62.5 | 47.5 |
| By Year |  |  |  |
| 2004 | 130 | 519 | 1349 |
| 2005 | 132 | 414 | 1458 |
| 2006 | 98 | 385 | 1419 |
| 2007 | 106 | 392 | 1647 |
| 2008 | 151 | 298 | 1785 |
| 2009 | 137 | 600 | 1733 |
| 2010 | 135 | 298 | 1689 |
| 2011 | 138 | 325 | 1960 |
| 2012 | 135 | 271 | 2300 |
| 2013 | 177 | 246 | 1781 |
| By group |  |  |  |
| in uveitis cohort | 98 | 68 | 233 |
| in control cohort | 42 | 258 | 944 |
| in uveitis cohort  (after the day of onset) | 59 | 31 | 106 |
| in control cohort  (after the index day) | 26 | 120 | 601 |

^*^Ankylosing spondylitis

^†^Rheumatoid arthritis

^‡^Herniated intervertebral disc

^§^Incidence rate per 100,000 person-years
