# Supplementary material for: Recurrent anterior uveitis and subsequent incidence of ankylosing spondylitis: a nationwide cohort study from 2002 to 2013
Source: Arthritis Res Ther. 2018 Feb 7;20:22. doi: 10.1186/s13075-018-1522-2 (PMC5804077; doi:10.1186/s13075-018-1522-2)
Supplement: Supplementary file 3 — Estimation of incidence rate ratio of ankylosing spondylitis in recurrent uveitis along with the time interval used for defining uveitis recurrence. (DOCX 13 kb) [file 13075_2018_1522_MOESM3_ESM.docx]

**Additional file 3: Table S3. Estimation of incidence rate ratio of ankylosing spondylitis in recurrent uveitis along with the time interval used for defining uveitis recurrence.**

| Time interval (days)^*^ | Recurred uveitis | Recurrent uveitis group^†^ | Subsequent AS case | Incidence rate | Incidence rate ratio (95% CI) |
| --- | --- | --- | --- | --- | --- |
| 90 | 2180 | 2168 | 21 | 255.9 | 16.33 (9.63–27.67) |
| 100 | 2144 | 2132 | 21 | 261.7 | 16.79 (9.90–28.49) |
| 110 | 2086 | 2074 | 21 | 270.5 | 17.32 (10.21–29.39) |
| 120 | 2050 | 2038 | 21 | 277.3 | 17.71 (10.44–30.06) |

^*^ Recurrence of uveitis was defined as claims of anterior uveitis with the treatment separated at least time interval criteria

^†^Among recurred uveitis, patients having AS before uveitis recurrence were excluded from recurrent uveitis group.
